# Socioeconomic and geographical disparities in disability distribution among Tanzanian population: insights from the 2022 demographic and health survey and malaria indicator survey

**DOI:** 10.1186/s12889-025-23152-x

**Published:** 2025-07-14

**Authors:** Pankras Luoga, Gladys Reuben Mahiti, Oliva Kapinga, Blandina Herzon, Syabo M. Mwaisengela, Patricia A. Materu, Chrisogone J. German, Amalberga Alex Kasangala, Grace Edward Mtulo, Erick S. Kinyenje, Joseph C. Hokororo, Teresia Lipyana Ngonyani, Godfrey Kacholi, Ntuli A. Kapologwe, Claud J. Kumalija, Mackfallen G. Anasel, Eliudi S. Eliakimu

**Affiliations:** 1 Department of Development Studies, School of Public Health and Social Sciences, Muhimbili University of Health and Allied Sciences, Dar es Salaam, United Republic of Tanzania; 2 School of Nursing, St. Johns University, Dodoma, United Republic of Tanzania; 3 Health Quality Assurance Unit, Ministry of Health, Dodoma, United Republic of Tanzania; 4 School of Nursing and Public Health, University of Dodoma, Dodoma, United Republic of Tanzania; 5 Department of Curative Services, Ministry of Health, Dodoma, United Republic of Tanzania; 6 Ministry of Community Development, Gender, Elderly and Children, Dodoma, United Republic of Tanzania; 7 Regional Health Management Team, Mtwara, United Republic of Tanzania; 8 Department of Health Systems Management, Mzumbe University, Morogoro, United Republic of Tanzania; 9 East, Central and Southern Africa– Health Community (ECSA– HC), Arusha, United Republic of Tanzania; 10 Health Monitoring and Evaluation Unit, Ministry of Health, Dodoma, United Republic of Tanzania

**Keywords:** Socioeconomic, Geographical disparities, Disability, Population, Demographic and health survey, Tanzania

## Abstract

**Background:**

Disability is a significant public health issue globally, especially in sub-Saharan Africa, including Tanzania, where its prevalence is high. Effective health service planning requires reliable data on disability prevalence and distribution. However, Tanzania lacks sufficient empirical data, hindering social service planning. This study aims to examine socioeconomic and geographical disparities in disability among Tanzanians aged 15–95 years.

**Methodology:**

This study utilized secondary data from the 2022 Tanzania Demographic and Health Survey and Malaria Indicator Survey (TDHS-MIS), comprising a weighted sample of 58,425 individuals. The dependent variable was disability status, while independent variables included demographic and socioeconomic factors. Descriptive analysis, chi-square tests, and modified Poisson regression were employed to assess associations, with *p*-values < 0.05 indicating significance.

**Results:**

The overall disability prevalence was 11%. Older individuals (≥ 50 years) had a higher prevalence ratio (Adjusted prevalence ratio (APR): 3.85; 95% CI: 3.33, 4.52) compared to those aged 15–29. Females had a higher prevalence ratio (APR: 2.67; 95% CI: 2.63, 2.71) than males. Individuals with secondary education (APR: 2.58; 95% CI: 2.51, 2.65) and separated/widowed individuals (APR: 2.91; 95% CI: 2.74, 3.09) were more likely to report disability. Wealthiest households (APR: 2.74; 95% CI: 2.75, 2.9) and those with health insurance (APR: 2.83; 95% CI: 2.86, 2.99) had higher disability prevalence. Regional disparities showed the Northern zone had a higher prevalence ratio (APR: 2.77; 95% CI: 2.87, 3.00) compared to the Lake zone.

**Conclusion:**

This study highlights significant disability prevalence in Tanzania, with older age, female gender, higher education, wealth, and marital separation as key risk factors for disability. The counterintuitive findings on wealth and education may indicate improved access to diagnosis. Regional disparities warrant further investigation. These findings emphasize the need for targeted interventions and further research into underlying mechanisms to improve support systems for vulnerable populations.

## Background

Globally, disability has been a major public health burden to individual, society and the nation at large. The burden is reported in both developed and developing countries [1–4]. This has been limiting individuals’ ability to enjoy their lives and participate in economic activities as well as limiting caregivers from going out to look for daily bread. For instance, the latter commonly use a significant percentage of their time to care for the disabled at home [5]. The disabilities can be of various forms, including hearing, seeing, communicating, walking, and mental disabilities [4, 6]. These disabilities can affect individuals at any age, from childhood to adulthood. In many cultures, disability is associated with bad luck and other related myths of misfortune [7, 8]. This stigma leads to mistreatment and deprives people with disabilities of important opportunities to enjoy life. Such trends are particularly pronounced in economically limited settings, especially developing countries [4, 9].

In sub-Saharan Africa, the burden of disabilities is a public concern at the average of 3.2% [10], has been linked with human immunodeficiency virus (HIV) whereby people living with HIV have high prevalence of disabilities such as: limitations of mobility and motor function, arthritis, visual impairment, hearing impairment, dementia, and developmental delays (motor delays) in children [10, 11]. This burden is exacerbated by limited resources, inadequate inclusive infrastructures, family conflicts, restricted treatment opportunities, cultural beliefs, and a shortage of professionals equipped to address disabilities [7, 8, 11, 12]. For instance, a study conducted in Sierra Leone reports that disabled women have limited access to maternal and reproductive health services, including family planning contributed by socioeconomic inequality [13]. Another study indicates that children with disabilities faces challenges in accessing health services “due to poverty, low education, inadequate healthcare systems, and shortage of healthcare professionals*”* [12].

In Tanzania, the prevalence of disabilities is a public concern [14–16]. Estimated based on “Household Budget Survey*”* in the fiscal year 2017/2018 in Mainland and 2019/2020 in Zanzibar have shown that “over 3.3 million people are living with disability” in which the prevalence is 6.8% for Mainland Tanzania and 3.2% in Zanzibar [15]. Another study by Carraro based on National Panel survey of 2021 has shown the prevalence to be 2.7% [17]. Some studies have estimated the burden at sub-national level (regions and local government authorities). For example, Dewhurst and colleagues in 2012 in Hai they found that the age-adjusted prevalence of severe disability was 3.7% and the age-adjusted prevalence of moderate disability was 6.2% [18]. Quinones and colleagues in a study in Mbeya and Iringa regions in 2021 found that “14% of adolescents were having disabilities” [19]. Sabariego and colleagues in 2024 have noted the importance of advocates, policy makers, statistical offices and country authorities to follow reliability and validity standards when recommending tools for disability data collection, in order to avoid inconcistencies in estimation disability prevalence [20]. In some societies, disabilities have been linked to abuse. For example, Tanzania has witnessed disabled children being locked inside homes and not being sent to school, thereby being denied their right to education [21, 22]. In other areas, parents, particularly fathers, have neglected the care of their disabled children [23, 24]. This situation has led to negative effects on both disabled children and society at large. The adverse impacts on disabled children include an inability to learn important life skills, missing opportunities to recognize and develop their talents, and lacking access to education [22]. Disability can also arise in middle age or during participation in various productive economic activities, while another type typically occurs in older age. All these factors adversely affect the health and economic status of individuals, families, and society as a whole. During the Coronavirus disease 2019 (COVID-19) pandemic, people with disabilities in Dodoma Region Tanzania faced challenges in access to water, sanitation and hygiene (WASH) services and other interventions to protect themselves, in which “people with mobility, hearing, and vision impairments were mostly affected [25]. Also, a paper by Mnyanyi recommended the Government, policymakers, and researchers should invest in intervention services for people with disabilities to enhance their participation and contribution to socio-economic development [26]. Children with disabilities in Tanzania faces a number of challenges including: challenge to access, lack of resources, lack of specialized centres, lack of data and research, cultural discrimination, and poor communication between health services providers and care givers [26].

To ensure proper planning and implementation of inclusive social services, including health, education, and economic opportunities for the subpopulation [14], reliable data on the burden is crucial. Evidence-based data are expected to provide several benefits to the community, including opportunities for the disabled population to learn essential life skills that will enable them to integrate into society, recognize their talents, and develop them to assist in various economic activities. Training caregivers and guardians of disabled individuals on how to provide care and support in their homes and society at large is also important. This presents an opportunity to design interventions that stimulate economic growth by increasing the participation of disabled individuals in economic activities while also allowing caregivers more time for their own economic pursuits. However, to date, limited empirical studies have assessed the prevalence and socioeconomic and geographical disparities in the distribution of disabilities among the general population in Tanzania. This study aims to fill that gap by determining the national prevalence and socio-economic and geographical disparities in the distribution of disabilities among Tanzanian population.

We used social disability theory to guide our conceptualisation, analysis and discussion of the study [27]. The contextual barriers that hinder the fulfillment of individual potentials among individuals include unsupportive environments. For example, inadequate infrastructure and policies for inclusive education [21], financial, cultural and educational barriers. This may include age, sex, educational level, being covered by health insurance or not [28, 29].

## Methods

### Study design and setting

This study used secondary data of a weighted sample of 58,425 people obtained from the 2022 Tanzania Demographic and Health Survey and Malaria Indicator Survey (TDHS-MIS). The survey used a two-stage stratified sampling method. The first stage involved the stratification of the clusters in rural/urban settings based on the most current census. The second stage involved clustering and, from the clusters, a selection of households to be interviewed during the survey. A detailed description of the methodology and questionnaires used in the survey is available in the final report of the 2022 TDHS-MIS [30]. The unit of analysis for this study was individuals aged 15–95 years at the time of the survey.

### Variables and measurement

#### Dependent variable

The disability status of the general population in Tanzania was assessed as the dependent variable. The responses were captured from the following DHS questions based on the Washington Group questions on disability [31]. Would you say that (name of household member) has no difficulty seeing, some difficulty, a lot of difficulty, or cannot see at all? Would you say that (name of household member) has no difficulty hearing, some difficulty, a lot of difficulty, or cannot hear at all? Would you say that (name of household member) has no difficulty understanding or being understood, has some difficulty, or has a lot of difficulty or cannot communicate at all? Would you say that (name of household member) has no difficulty remembering or concentrating, some difficulty, a lot of difficulty, or cannot remember or concentrate at all? Would you say that (name of household member) has no difficulty walking or climbing steps, some difficulty, a lot of difficulty, or cannot walk or climb steps at all? Would you say that (name of household member) has no difficulty washing all over or dressing, some difficulty, a lot of difficulty, or cannot wash all over or dress at all? Responses were as follows: 1 = No difficulty in that domain, 2 = some difficulty, 3 = a lot of difficulty, 4 = cannot perform/function at all in the domain [30].

A composite dependent variable was created using the six domains captured in the DHS, including seeing, hearing, communicating, remembering or concentrating, walking or climbing steps (locomotion), and washing all over or dressing. The dependent variable was re-categorized to combine those with some difficulties and those with a lot of difficulties to be have disability coded as “1” and no any difficulty to be “no difficulty’ coded as “0”. An individual who responded ‘yes’ in at least one of the six domains was categorized as having a disability. The binary variable was re-categorised into no/yes coded as “0” for no and “1” for yes. Cases with missing data on the dependent variable were excluded from the analysis.

#### Independent variables

The independent variables in this study included age (15–29, 30–49 and 50+), sex of household members (female/male), education level (no formal education, primary education, secondary and beyond education), marital status (never married, married, widowed/divorced), type of place of residence (urban/rural), wealth quintile (poorest, poorer, middle, richer and richest), sex of household head (female/male), health insurance coverage (no/yes) and geographical zones (Lake zone, Coast zone, Northern zones, Central zone and Southern zones). Some of these variables were recoded; age, education level and geographical zones, were re-categorised to match our study backed up by previous studies done on the area of the study. These variables have also been used in other similar studies elsewhere [4].

### Data analysis

Data preparation and management was conducted. Descriptive analysis was performed to indicate the distribution of the study respondents in various categories in frequency and percent. Bivariable analysis using Chi-square was conducted to determine the association between dependent and independent variables. In addition, a bivariable modified Poisson regression model was used to determine the crude prevalence ratio (CPR) between an independent variable and dependent variable. Before entering all variables to the Modified Poisson logistic regression model, we tested for multi-collinearity to establish any collinearity among independent variables. In this case, there was no variable found to have collinearity to each other. Therefore, we entered all independent variables in the Modified Poisson regression model to determine the magnitude of association between categories of independent and dependent variables. Based on the literature, all potential confounders were controlled for by entering them into the Modified Poisson regression model. The threshold of *p*-value < 0.05 at 95% Confidence Interval (CI) was used to determine the significant factor. In addition, all analyses applied weight as recommended by the DHS programme [32].

## Results

### Socio demographic characteristics of the study respondents

The study involved a weighted sample of 58,425 people, of which more than a half (62.8%) were aged between 5 and 29 years with a mean age of 27.23 years. More than a half (53.1%) were females. More than a half (60.8%) had primary education, and more than a half (57.9%) were married. Almost three quarters (70.4%) were from rural settings. Less than a quarter (20.4%) were from the richest households. Among the sampled population, only 8.6% were covered by health insurance. Almost three quarters (74.8%) were male headed households. Almost a one-third (38.8%) were from the Lake zone, as detailed in Table 1.

**Table 1:** Socio-demographic characteristics of the study respondents in Tanzania using the 2022 demographic and health survey and malaria Indicator survey (*N* = 58,425)

Variable	Frequency (*N*)	Percent (%)
**Age of household members** (*N* = 58,364)		
15–29	36,681	62.8
30–49	12,761	21.9
50+	8,922	15.3
**Sex of household members**		
Male	27,423	46.9
Female	31,002	53.1
**Educational level of members** (*N* = 58,330)		
No formal education	12,136	20.8
Primary education	35,492	60.8
Secondary and beyond	10,702	18.3
**Marital status (N =** 37,554)		
Never married	10,108	26.9
Married	21,759	57.9
Widowed/divorced	5,687	15.1
**Type of place of residence**		
Urban	17,304	29.6
Rural	41,121	70.4
**Wealth index for urban/rural**		
Poorest	11,372	19.5
Poorer	11,517	19.7
Middle	11,733	20.1
Richer	11,860	20.3
Richest	11,943	20.4
**Covered by health insurance (** ***N*** ** = 29,** **794)**		
No	27,145	91.1
Yes	2,550	8.6
Don’t know	100	0.3
**Sex of household head**		
Male	43,719	74.8
Female	14,706	25.2
**Geographical zones**		
Lake zones	22,672	38.8
Northern zone	6,833	11.7
Central zone	6,703	11.5
Southern	11,760	20.1
Coast zone	10,457	17.9
**Disability combined**		
No difficulty	51,981	89.0
Yes have difficulty	6,444	11.03

### Geographical distribution of disabilities in Tanzania

**Fig. 1:**
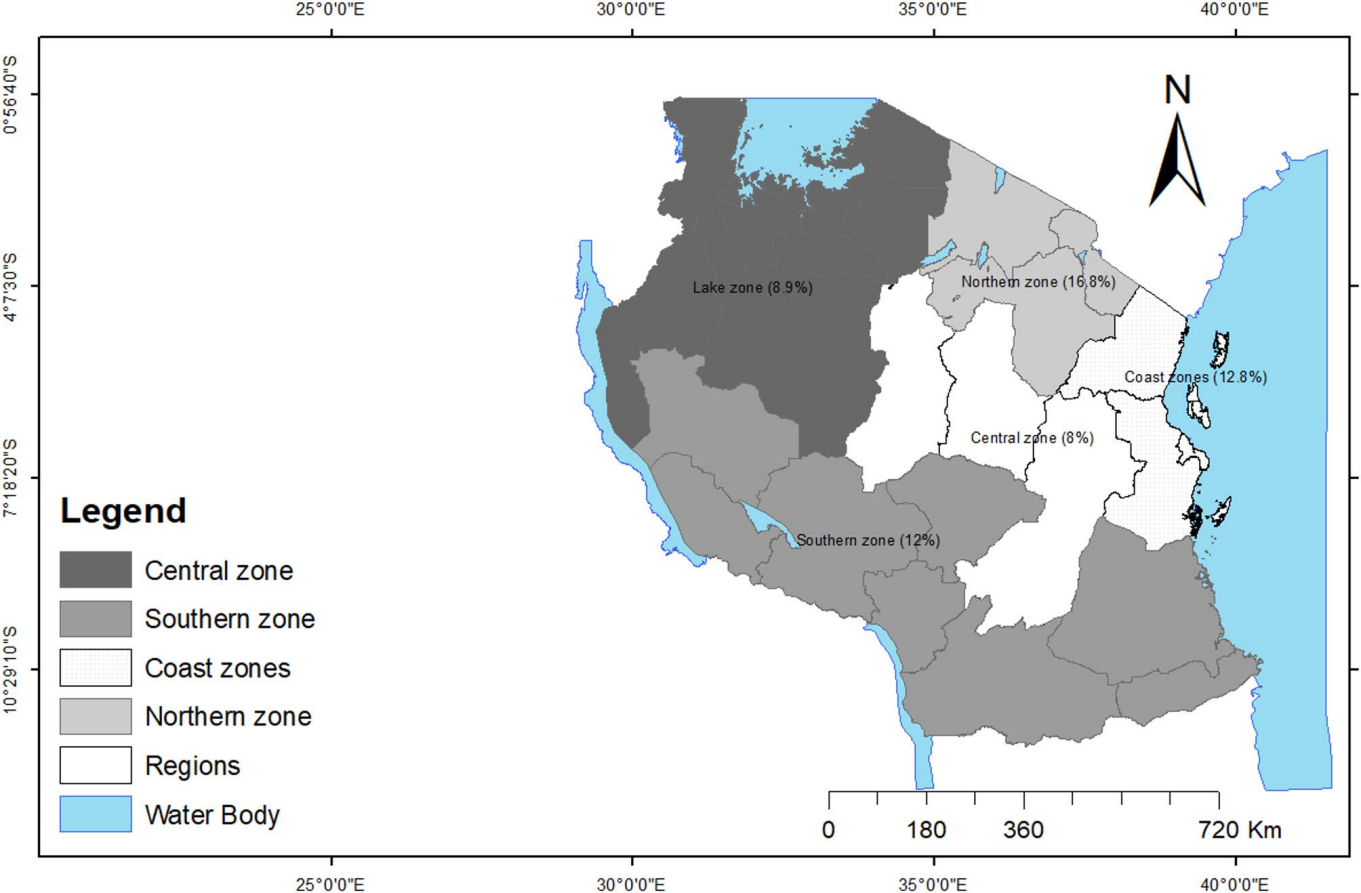
A map showing distribution of disabilities across geographical zones in Tanzania

The geographical distribution of disabilities in Tanzania shows the highest prevalence (16.8%) in the Northern zone and the lowest (8%) in the Central zone. As indicated in the map Fig. 1.

### The major types of disabilities among Tanzanian population aged 15–95 years

**Fig. 2:**
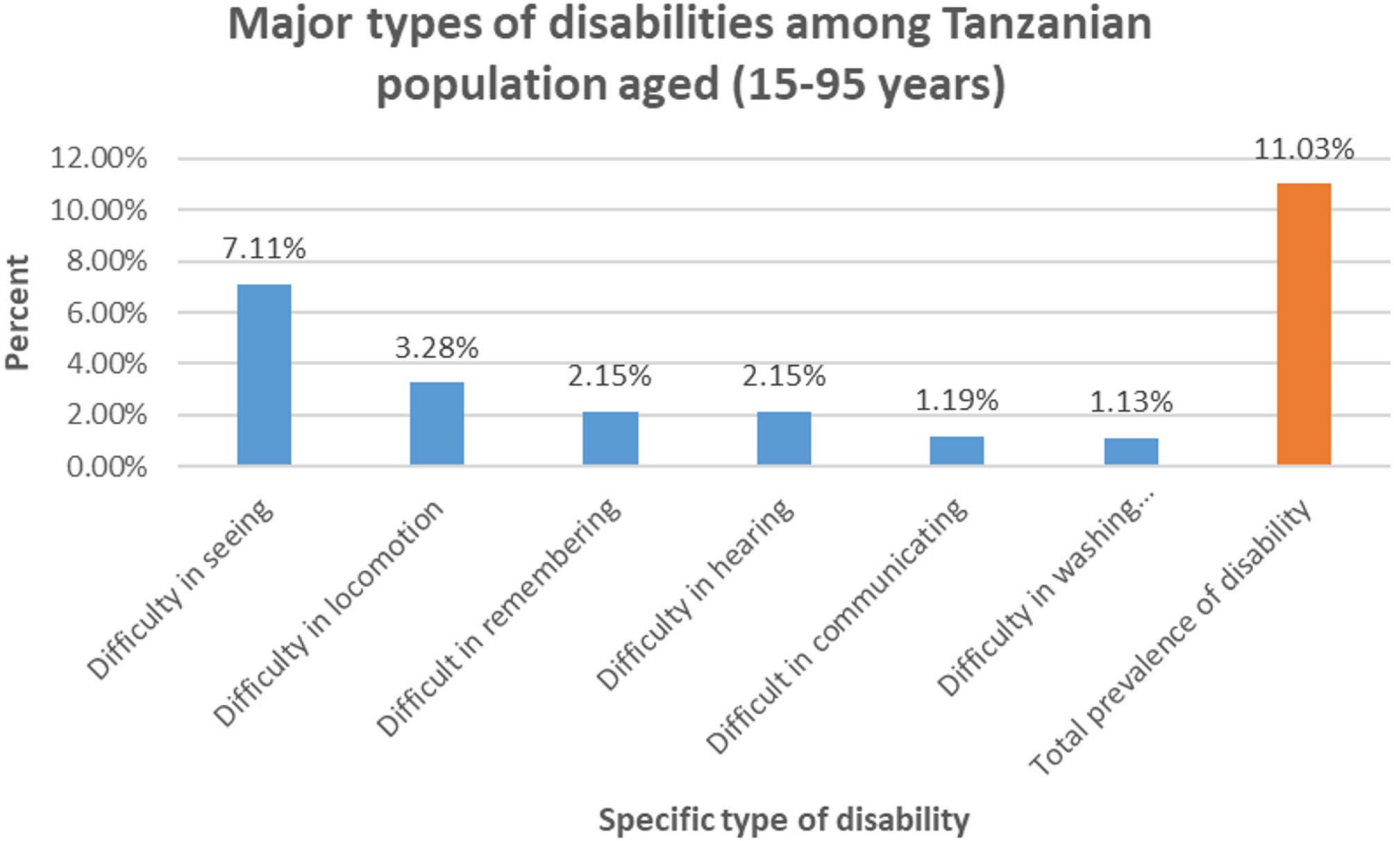
Showing major types of disabilities among the Tanzanian population aged 15–95 years

Figure 2 shows the major types of disabilities in Tanzania. The overall prevalence of disability status (11.03%) was determined by number of persons with at least one disability. The leading (7.11%) is difficulty in seeing followed up by difficulty in the ability to make movement or the ability to move from one place to another (locomotion) and the lowest (1.13%) is difficulty in washing all over or dressing.

### The socioeconomic and geographical disparities in disability among the Tanzanian population from the chi-square model

Among the population aged 15–29, 4.4% had disability; among the population aged 30–49, 11% had disability; and among the population aged 50 and above, 38% had disability. Among male and female, 10% and 11.9% respectively had disability. Among the population with no formal education, 15.1% had disability; among the population with primary education, 10.2% had disability; and among the population with secondary and above education 9 0.3% had disability. Among those who are never married, 7.9% had disability; among those who are married, 14% had disability; and among those who are separated/widowed, 32% had disability. Among the urban population, 12.5% had disability; and 10.4% of the rural population had disability. Among the individuals from poorest households, 9.5% had disability; among those from poorer households, 11.7% had disability; among those from middle economic status households, 10.5% had disability; among those from richer economic status households,10% had disability; and among those from richest households 13.3% had disability. Also, among those who are not covered by health insurance, 12.5% had disability; among those who are covered by health insurance, 29.8% had disability; and among those who don’t know whether they are covered by health insurance or not, 20.1% had disability. Among those who are coming from male headed households, 9.9% had disability; and among those who are coming from female headed households, 14.4% had disability. Among those who are coming from Lake zone, 8.9% had disability; among those who are coming from Northern zone, 16.8% had disability; among those who are coming from Central zone, 8% had disability; and among those who are coming from Southern zone, 12% had disability; among those who are coming from Eastern zone, 12.8% had disability. All factors included in the model were significantly associated with disability status, as detailed in Table 2.

**Table 2:** Bivariate analysis between independent and dependent variables in Tanzania using the 2022 demographic and health survey and malaria Indicator survey

Variable	General disability			
	Have difficulty			
	%	(CI)	Chi-Square (X^2^)	*p*-value
**Age of members of a household**			8414.5234	< 0.001
15–29	4.4	[4.2,4.8]		
30–49	11.0	[10.4,11.8]		
50+	38.0	[36.6,39.3]		
**Sex of household member**			51.9206	< 0.001
Male	10.0	[9.6,10.5]		
Female	11.9	[11.3,12.5]		
**Educational level members household**			274.7630	< 0.001
No formal education	15.1	[14.1,16.2]		
Primary education	10.2	[9.7,10.7]		
Secondary and beyond	9.3	[8.5,10.1]		
**Marital status**			1727.2382	< 0.001
Never married	7.9	[7.3,8.6]		
Married	14.0	[13.2,14.8]		
Widowed/divorced	32.0	[30.4,33.5]		
**Type of place of residence**			52.0837	< 0.001
Urban	12.5	[11.7,13.3]		
Rural	10.4	[9.9,11.]		
**Wealth index for urban/rural**			109.7606	< 0.001
Poorest	9.5	[8.7,10.4]		
Poorer	11.7	[11.,12.6]		
Middle	10.5	[9.7,11.4]		
Richer	10.0	[9.3,10.8]		
Richest	13.3	[12.1,14.6]		
**Covered by health insurance**			604.6994	< 0.001
No	12.5	[11.9,13.1]		
Yes	29.8	[27.1,32.8]		
Don’t know	20.1	[11.6,32.4]		
**Sex of head of household**			230.8242	< 0.001
Male	9.9	[9.4,10.4]		
Female	14.4	[13.6,15.2]		
**Geographical zones**			452.5690	< 0.001
Lake zones	8.9	[8.2,9.7]		
Northern zone	16.8	[14.9,18.8]		
Central zone	8.0	[6.9,9.3]		
Southern zone	12.0	[11.3,12.8]		
Coast zone	12.8	[11.9,13.8]		

### Disparities in the prevalence of disability among the Tanzanian population from the modified Poisson regression model

After controlling for other covariates, the older population aged 50 and above had (APR; 3.85;95%CI: 3.33, 4.52, *p* < 0.001) a higher prevalence ratio of having disability compared to the younger population aged below 30 − 19. Females had (APR; 2.67; 95% CI: 2.63, 2.71, *p* = 0.034) higher prevalence ratio of having disability compared to males. Individuals with secondary and beyond education level (APR: 2.58; 95% CI: 2.51, 2.65, *p* < 0.001) and individuals with primary education (APR: 2.62; 95% CI: 2.58,2.66, *p* < 0.001) had a higher prevalence ratio of having disability compared to those who did not have formal education. Separated/widowed individuals had (APR; 2.91; 95% CI: 2.74, 3.09, *p* < 0.021) higher prevalence ratio of having disability compared to those who are never married. Individuals from the richest households had (APR; 2.74; 95%CI: 2.75,2.90, *p* < 0.003) higher prevalence ratio of having disability compared to those who are from poorest households. Individuals who are covered by health insurance had (APR; 2.83; 95%CI: 2.86, 2.99, *p* < 0.001) higher prevalence ratio of having disability compared to those who are not covered by health insurance. Individuals from the Northern zone had (APR; 2.77; 95% CI: 2.87, 3.00, *p* < 0.001) higher prevalence ratio of having disability compared to those who are from the Lake zone. However, the type of place of residence and the sex of the household head were not significantly associated with the risk of having disability, as indicated in Table 3.

**Table 3:** Modified Poisson regression on the socioeconomic and geographical factors associated with the disability among population aged 5–95 years in Tanzania using 2022 demographic and health survey and malaria Indicator survey

Variable	**Disability**					
	CPR	CI	*P*-value	**APR**	CI	*P*-value
**Age**						
15–29	Ref			Ref		
30–49	1.06	[1.06, 1.07]	< 0.001	1.62	[0.49, 2.76]	0.097
50+	1.31	[1.13, 1.33]	< 0.001	3.85	[3.33, 4.52]	< 0.001
**Sex of household members**						
Male	Ref			Ref		
Female	1.02	[1.01,1.02]	<0.001	2.67	[2.63, 2.71]	0.034
**Educational level**						
No formal education	Ref			Ref		
Primary education	0.95	[0.94, 0.96]	< 0.001	2.62	[2.58,2.66]	< 0.001
Secondary and beyond	0.94	[0.94,0.95]	< 0.001	2.58	[2.51, 2.65]	< 0.001
**Current marital status**						
Never married	Ref			Ref		
Married	1.065	[1.06, 0.07]	< 0.001	2.62	[2.49, 2.76]	0.168
Separated or widowed	1.23	[1.22,1.24]	<0.001	2.91	[2.74,3.09]	0.021
**Type of place of residence**						
Rural	Ref			Ref		
Urban	1.02	[1.01,1.02]	< 0.001	2.76	[2.71, 2.81]	0.083
**Wealth index for urban/rural**						
Poorest	Ref			Ref		
Poorer	1.01	[1.00, 1.02]	< 0.003	2.83	[2.77, 2.90]	< 0.001
Middle	1	[0.99, 1.01]	0.844	2.78	[2.71, 2.84]	0.078
Richer	1	[1.00, 1.01]	0.334	2.78	[2.67,2.80]	0.57
Richest	1.02	[1.02, 1.03]	< 0.001	2.74	[2.75,2.90]	0.003
**Health insurance**						
No	Ref			Ref		
Yes	1.13	[1.12, 1.15]	<0.001	2.83	[2.86, 2.99]	< 0.001
Don’t know	1.09	[1.02, 1.17]	0.008	2.93	[2.48, 3.01]	0.976
**Sex of household heard**						
Male	Ref			Ref		
Female	1.04	[1.04,1.05]	< 0.001	2.72	[2.71, 2.83]	0.073
**Geographical zones**						
Lake zone	Ref			Ref		
Northern zone	1.08	[1.07,1.09]	< 0.001	2.77	[2.87, 3.00]	< 0.001
Central zone	1	[0.99,1.00]	0.48	3.11	[2.81, 2.93]	0.021
Southern	1.03	[1.03,1.04]	< 0.001	2.64	[2.58, 2.71]	< 0.001
Coast zone	1.04	[1.03,1.04]	< 0.001	2.87	[2.87, 3.00]	<0.001

Keys:

APR = Adjusted Prevalence Ratio

CPR = Crude Prevalence Ratio

CI = Confidence Interval

## Discussion

The study aimed to determine the prevalence of disability among the general population in Tanzania. The overall prevalence of disability in the study setting is 11.03%. Specifically, the major types of disabilities within the Tanzanian population are difficulty in seeing at 7.11%, followed by difficulty in movement (the ability to move from one place to another) and the lowest prevalence, at 1.13%, being difficulty in washing or dressing.

Our analysis showed that the older population aged 50 and above had three times the risk of having a disability compared to the younger population aged between 5 and 30. This may imply that older age is a risk factor for developing some of the disabilities like not able to see, to remember and walk. This finding contradicts those reported elsewhere, where older individuals were less likely to report having a disability [33]. However, the explanations provided by the study are understandable given the sufficient public services offered to the elderly in those settings [33]. This may contrast with the situation in Tanzania, where the elderly are said to be entitled to free services but still express complaints [34, 35]. This finding align with social disability model whereby age is indicated as barriers to fulfilment of individual potentials.

Furthermore, individuals with secondary and higher education levels had a higher prevalence ratio of having a disability compared to those with no formal education. This has no a clearly established linkage. However, a possible explanation could be that difficulties in seeing and the need for glasses may classify many educated people, who typically experience challenges in reading or seeing, as disabled. Perhaps could be due to reporting bias as educated people, who are more likely to notice and report small vision problems since they read more often, are classified as disabled based on that, it could inflate disability prevalence among the subgroup.

Our analysis found that individuals from the richest households had almost two times higher prevalence ratio of having disability compared to those who are from poorest households. In Tanzanian context, this could imply differences in health seeking behaviors that individuals from richest households may access health services than individuals from the poorest households, particularly those with disabilities, face significant challenges in accessing healthcare services due to the high costs associated with managing disability conditions [36]. When they access health services more than individuals from poor population, this leads to more rich people to be diagnosed with disability compared to poor individuals who may rarely access health services because they are not able to cover the cost of care including getting health insurance or exemption [35, 37, 38]. This is contrary to the finding reported in India whereby prevalence of disability was reported to be higher among poorest households [4].

Those individuals who are covered by health insurance had two times higher risk of having disabilities compared to those who are not covered by health insurance. This may imply the presence of adverse selection in the enrollment of members in the voluntary health insurance schemes like improved community Fund (iCHF) and voluntary packages including “Afya Toto Kadi” in National Health Insurance Fund (NHIF) in the country. This is the tendency of many people to enroll in the health insurance scheme only after recognising that they have a disability or chronic health condition requiring regular check-up or ongoing treatment [39], or for a case of household that has an elder individual (above 60 years) which has been shown to influence their enrollment in iCHF [40]. This may lead to occurrence of statistics that show being covered by health insurance is linked to higher disability prevalence [41]. A similar finding was reported in a study conducted among older adults in Ghana, which found that those without health insurance were less likely to have disability conditions [42] and insured individuals having higher possibility of seeking for health care than uninsured individuals [41].

Female-headed households had two times higher risk of having disabilities compared to male-headed households. This may imply living in a female headed household increases likelihood of obtaining disability caused by poor quality health services and sometimes even not receiving treatments services. This finding is similar to the findings reported elsewhere, whereby female-headed households were more likely to report having a disability compared to male-headed households [33].

The Northern zone had almost thrice times the prevalence ratio of having disabilities compared to the Lake zone. This may suggest that there is a need for further research to understand the underlying contributing factors. Nevertheless, potential reasons could be stigma and cultural beliefs toward disabled people in the setting, making communities not to seek for healthcare including some conditions that can be corrected through early could medical intervention, surgery, therapy, or assistive devices. Also, the zone appears to require targeted attention given the fact that some studies in the Kilimanjaro Region (one of the regions making up the northern zone) have pointed out that people with disability face difficulties in accessing needed services such as rehabilitation services and schools [36, 43], including adaptations in “*higher-learning institutions*” [44]. A need for a “sustainable support system” for families caring for people with disability in that region have been raised [45].

Moreover, marital status showed to have association with disability conditions. Specifically, separated or widowed individuals had two time a higher prevalence of disability compared to individuals who have never married. In Tanzanian context, this may potentially be due to situations occurred that result to separation or widow status including death of a spouse, disputes including domestic violence and abuse, poverty and economic stress [46–48]. The specific aforementioned situations are likely to lead injuries, stigma, isolation, HIV risks, mental health issues and stress [49].

The reported findings in the current study align with social disability model whereby age, wealth index, health insurance coverage, and geographical zone were indicated as barriers to fulfilment of individual potentials among disabled population.

### Strengths and limitations of the study

The main strength of the 2022 TDHS-MIS data lies in its comprehensive representation of the disability population across the nation, as well as its nested data structure. This unique structure enabled the current study to determine socioeconomic and geographical disparities related to disabilities within the Tanzanian demographic, specifically among individuals aged 15 to 95 years. Furthermore, the quality of DHS data which is collected by the use of standard questionnaires used in more than 90 countries is acknowledged. This strength enhances the external validity of the findings and provides a comparative advantage with other countries where the DHS is also used. However, the cross-sectional nature of the data used in the analysis limited the establishment of a causal-effect relationship. This is because the independent and dependent (outcome) variables were collected at one point in time. In addition, the TDHS did not capture cultural factors (for instance beliefs towards the occurrence of disability in the family) which are well documented to be linked with disabilities [7, 8]; hence, the current analysis inherits this limitation.

## Conclusion

The general prevalence of disability among the Tanzanian population is 11.03%. This study reveals a substantial disability prevalence in Tanzania, highlighting the vulnerability of older individuals, females, those with secondary education, separated/widowed individuals, and the wealthiest. The counterintuitive association with higher education and wealth may reflect improved access to diagnosis and reporting. Regional disparities warrant further investigation, particularly the higher prevalence in the Northern zone. These findings underscore the need for developing relevant interventions to address the complex factors contributing to disability and improve support systems, especially for vulnerable populations. Further research to determine the factors associated with specific disability conditions and qualitative study to explore the mechanisms underlying these associations are recommended.

### Policy implications

The study highlights findings that could inform political leaders, policy and decision makers on areas of focus as Tanzania continue to implement the Sustainable Development Goals (SDGs) 2030 in the second half of the SDGs era; 2015–2030, which is marked with efforts to recover from the COVID-19 [50]; and address other crises including climate change effects such as floods and landslide [51] and emerging and reemerging infectious disease outbreaks such as leptospirosis [52, 53], Marburg virus disease [54] and Cholera [55]. The revised version of the Tanzania Development Vision 2025 [56], the “*Draft Tanzania Development Vision 2050*” with 20-high level targets including targets that address “extreme poverty; universal high quality health, water and social protection; attainment of universal secondary education and 15% attaining higher education” [57], should guide everyone in ensuring that multi-sectoral interventions are put in place to address the burden of disability using the findings from this study that has utilized the data from 2022 TDHS-MIS. A need for a “sustainable support system” for families caring for people with disability in Kilimanjaro region has been raised [45], which implies that there is a need for localized contextual interventions based on available data and evidence in the specific geographical zones. Furthermore, the Northern zone have higher number of aged population that can indicate availability of relatively disabilities linked to the aged population themselves [58].

## Data Availability

The data used in the analysis are available online and can be requested from the DHS website (https://dhsprogram.com/).
